# Influenza Viruses Suitable for Studies in Syrian Hamsters

**DOI:** 10.3390/v14081629

**Published:** 2022-07-26

**Authors:** Shufang Fan, Chunyang Gu, Huihui Kong, Lizheng Guan, Gabriele Neumann, Yoshihiro Kawaoka

**Affiliations:** 1 Department of Pathobiological Sciences, School of Veterinary Medicine, University of Wisconsin-Madison, Madison, WI 53711, USA; shufang.fan@wisc.edu (S.F.); chunyang.gu@wisc.edu (C.G.); huihuikong1987@yahoo.com (H.K.); lizheng.guan@wisc.edu (L.G.); gabriele.neumann@wisc.edu (G.N.); 2 Division of Virology, Department of Microbiology and Immunology, Institute of Medical Science, University of Tokyo, Tokyo 108-8639, Japan; 3 Research Center for Global Viral Diseases, National Center for Global Health and Medicine, Tokyo 162-8655, Japan

**Keywords:** influenza virus, replication, Syrian hamster

## Abstract

Several small animal models, including mice, Syrian hamsters, guinea pigs, and ferrets are used to study the pathogenicity, transmissibility, and antigenicity of seasonal and pandemic influenza viruses. Moreover, animal models are essential for vaccination and challenge studies to evaluate the immunogenicity and protective efficacy of new vaccines. However, authentic human influenza viruses do not always replicate efficiently in these animal models. Previously, we developed a high-yield A/Puerto Rico/8/34 (PR8-HY) vaccine virus backbone that conferred an increased virus yield to several seasonal influenza vaccines in eukaryotic cells and embryonated chicken eggs. Here, we show that this PR8-HY genetic backbone also increases the replication of several seasonal influenza viruses in Syrian hamsters compared to the authentic viruses. Therefore, the PR8-HY backbone is useful for animal studies to assess the biological properties of influenza viral HA and NA.

## 1. Introduction

By escaping the existing immunity in humans, seasonal influenza viruses cause annual epidemics with increases in morbidity and mortality. Suitable animal models are essential to characterize the pathogenicity, transmission, and antigenicity of these viruses and to test novel vaccine preparations and vaccination approaches. Mice and ferrets are frequently used for influenza virus research, but Syrian hamsters [1,2,3] and guinea pigs [4,5,6] are also now well-established as small animal models. Mice are frequently used in influenza virus studies, but they are not a natural host of influenza viruses; human influenza A and B viruses may require adaptation to mice, which limits their use as an animal model in human influenza virus research (reviewed in [6,7]). Several mouse-adapted laboratory strains, such as A/Puerto Rico/8/34, A/WSN/33, and B/Lee/1940, are available, but these viruses are antigenically different from recent human influenza viruses. As an alternative, recombinant viruses that possess the HA and NA genes of a human influenza virus in the genetic background of PR8 have been used for studies in mice (reviewed in [6]). Ferrets, Syrian hamsters, and guinea pigs are susceptible to natural influenza virus isolates, but some viruses—especially recent human H3N2 viruses—do not replicate to high titers in these animals (our unpublished findings), which can present a limitation to certain studies, such as vaccination and challenge experiments.

Previously, we developed a high-yield A/Puerto Rico/8/34 (PR8-HY) influenza virus backbone that improved the titers of influenza vaccine viruses of different subtypes in mammalian cells and embryonated chicken eggs [8]. Here, we demonstrate that the PR8-HY virus backbone also increases influenza virus replication in Syrian hamsters, compared to authentic virus isolates.

## 2. Materials and Methods

### 2.1. Cells and Viruses

First, 293T human embryonic cells were purchased from the American Type Culture Collection (ATCC, Manassas, VA, USA) and maintained in Dulbecco’s Modified Eagle Medium (DMEM) containing 10% fetal bovine serum (FBS). Madin–Darby canine kidney (MDCK) cells expressing increased amounts of α2,6-linked sialic acid and decreased amounts of α2,3-linked sialic acid (dubbed humanized MDCK cells, hCK [9]) were maintained in minimal essential medium (MEM) containing 5% newborn calf serum at 37 °C in 5% CO_2_.

In this study, we used the following authentic human H3N2 and 2009 pandemic H1N1 (pdmH1N1) viruses: A/Wisconsin/15/2009 (Wisconsin/09, H3N2; kindly provided by the CDC; EPI_ISL_98964), A/Tokyo/IMS2-1/2014 (Tokyo/14, H3N2; kindly provided by the University of Tokyo; sequence not available in GISAID), A/Isumi/UT-KK001-1/2018 (pdmH1N1; kindly provided by the University of Tokyo; sequence not available in GISAID), A/Perth/16/2009 (Perth/09, H3N2; kindly provided by Dr. Ron Fouchier, Erasmus Medical Center, Rotterdam; EPI_ISL_2404688), A/Darwin/102/2019 (Darwin/19, pdmH1N1; kindly provided by Dr. Ian Barr, WHO Collaborating Center for Reference and Research on Influenza, Melbourne; EPI_ISL_380101), A/Idaho/07/2018 (Idaho/18, pdmH1N1; kindly provided by the CDC; EPI_ISL_305199), and A/Washington/23/2020 (Washington/20; kindly provided by the CDC; EPI_ISL_421133).

### 2.2. Virus Generation

Briefly, the HA and NA genes of the respective influenza viruses were cloned into the pHH21 vector for reverse genetics [10]. To generate recombinant viruses, the RNA polymerase I-controlled plasmids for HA, NA, and the six internal genes of PR8-HY virus [8] were co-transfected into 293T cells, together with four plasmids expressing the viral replication complex (PB2, PB1, PA, and NP) and one plasmid expressing human airway trypsin-like protease (HAT). The 293T supernatant was collected at 48 h post-transfection and the virus was amplified in hCK cells [9]. Virus stocks were sequenced before use.

### 2.3. Virus Replication in Syrian Hamsters

Eight- to ten-week-old male Syrian hamsters (Charles River, Wilmington, MA, USA) were used in this study. Per virus, six hamsters were intranasally inoculated with 10^6^ plaque-forming units (PFU) of the indicated viruses in a volume of 100–150 μL. This is based on our previous finding that an infection dosage of 10^6^ PFU and inoculation volume of 100 µL results in relatively low variability in virus titers, whereas an infection dosage of 10^5^ PFU and inoculation volume of 10 µL results in higher variability in virus titers because not all animals may get infected at the lower volume (Figure S1). On day 3 and 5 post-infection (p.i.), three Syrian hamsters from each group were euthanized and the nasal turbinates, tracheas, and lungs were collected for virus titration in hCK cells.

### 2.4. Statistical Analysis

Statistical analyses were performed by using Prism 8 software (GraphPad, San Diego, CA, USA). The unpaired *t*-test was used to compare viral titers in hamster tissue samples. A *p* value of less than 0.05 was considered significant.

## 3. Results

### 3.1. PR8-HY Internal Genes Enhance the Replication of H3N2 Viruses in Hamsters

**Figure 1:**
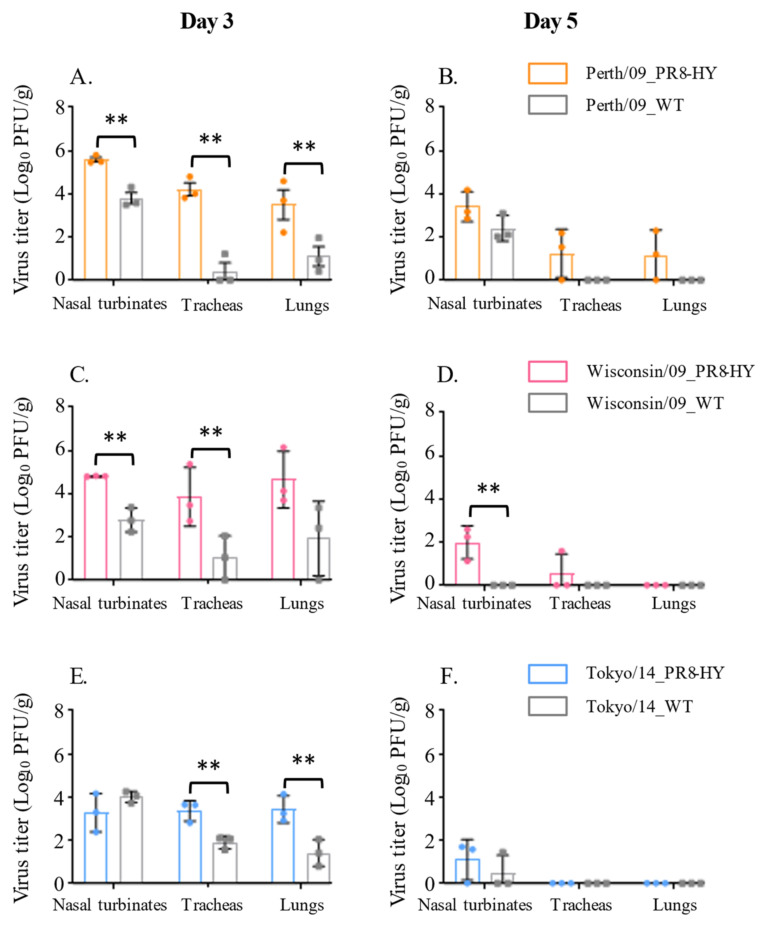
Replication of authentic and PR8-HY-based human H3N2 viruses in Syrian hamsters. Syrian hamsters (six per virus) were infected with 10^6^ PFU of wild-type isolate or recombinant virus with the respective HA and NA genes on the PR8-HY backbone. On days 3 and 5 p.i., three animals from each group were euthanized and virus titers in the nasal turbinates, tracheas, and lungs were determined in hCK cells; ** *p* < 0.01. (**A**,**C**,**E**) show the titers of the indicated viruses on day 3 post-infection; (**B**,**D**,**F**) show the titers of the indicated viruses on day 5 post-infection.

Authentic human influenza virus isolates may not replicate efficiently in small animal models. To test whether the PR8-HY genetic backbone [8] increases the replication of recombinant viruses with the HA and NA genes of contemporary human influenza viruses in Syrian hamsters, we generated three viruses possessing the H3N2 Perth/09, Wisconsin/09, and Tokyo/14 HA and NA genes, in combination with the remaining genes of PR8-HY virus and tested the titers of the authentic and PR8-HY recombinant virus stocks in hCK cells (i.e., Madin–Darby canine kidney (MDCK) cells expressing increased amounts of α2,6-linked sialic acid and decreased amounts of α2,3-linked sialic acid) [9]. All three recombinant PR8-HY viruses substantially increased the virus titer compared to the authentic viruses (Table 1). To compare the replication of authentic and recombinant viruses in Syrian hamsters, for each virus, we infected six Syrian hamsters with 10^6^ PFU of virus and collected nasal turbinates, tracheas, and lungs on days 3 and 5 post-infection. The virus titers of the samples were determined in hCK cells [9]. The PR8-HY backbone increased virus titers 10–100-fold in animals infected with Perth/09_PR8-HY and Wisconsin/09_PR8-HY on day 3 post-infection (Figure 1A,C), and facilitated virus replication on day 5 post-infection in most organs tested (Figure 1B,D). On day 3 post-infection, the PR8_HY backbone also significantly increased the virus titers in the tracheas and lungs of animals infected with the Tokyo/14_PR8-HY virus (Figure 1E). In the nasal turbinates, the PR8_HY backbone led to slightly lower titers on day 3 post-infection (Figure 1E), but increased titers on day 5 post-infection (Figure 1F). Generally, virus titers are higher on day 3 compared to day 5 post-infection, indicating virus clearance at the latter time point. Neither the authentic viruses nor the PR8-HY reassortant viruses caused significant weight loss or other clinical signs of disease. These data indicate that the PR8-HY backbone enhances virus replication in Syrian hamsters compared to the authentic human influenza virus. 

**Figure 2:**
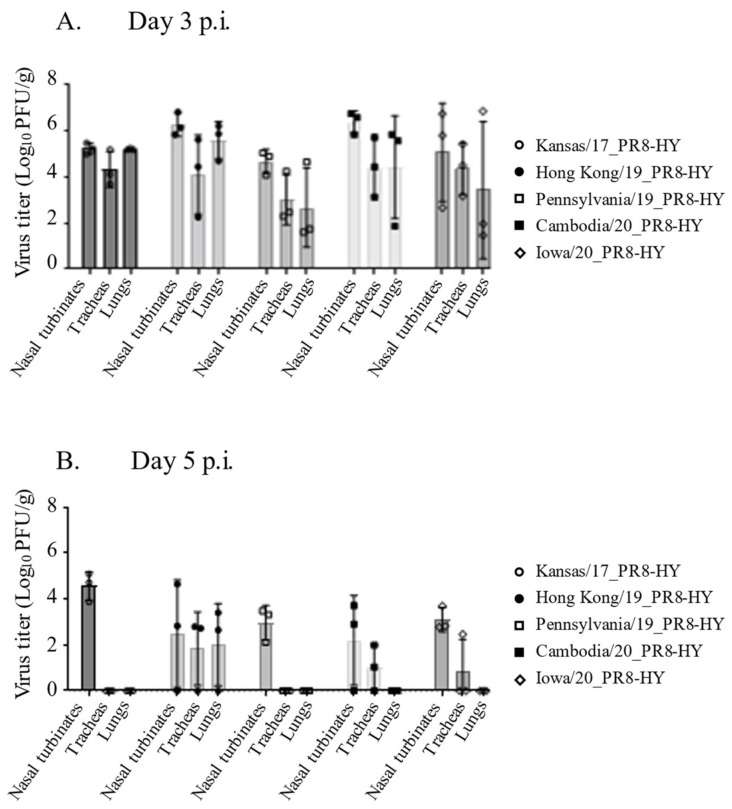
Replication of recombinant human H3N2 viruses in Syrian hamsters. Syrian hamsters (six per virus) were infected with 10^6^ PFU of recombinant virus with the respective HA and NA genes on the PR8-HY backbone. On days 3 and 5 p.i., three animals from each group were euthanized and virus titers in the nasal turbinates, tracheas, and lungs were determined in hCK cells.

To further assess the potential of the PR8-HY backbone for influenza studies in animal models, we next generated five recombinant viruses with the PR8-HY backbone and the HA and NA genes of the following recent H3N2 viruses representing different clades: A/Kansas/14/2017 (Kansas/17, clade 3c.3a; EPI_ISL_2454700), A/Hong Kong/45/2019 (Hong Kong/19, clade 3c.2a1b.1b; EPI_ISL_410589), A/Pennsylvania/1026/2019 (Pennsylvania/19, clade 3c.2a1b.1b; EPI_ISL_398842), A/Cambodia/e0829360/2020 (Cambodia/20, clade 3c.2a1b.2a.1; EPI_ISL_710475), and A/Iowa/13/2020 (Iowa/20, clade 3c.2a1b.1a; EPI_ISL_413744). All five recombinant viruses replicated to moderate-to-high titers in the upper and lower respiratory tracts of Syrian hamsters on day 3 post-infection, ranging from 2.7 to 6.4 log_10_ PFU/g (Figure 2A). On day 5 post-infection, all five viruses were isolated from the nasal turbinates of infected animals (Figure 2B). The recombinant Cambodia/20_PR8-HY, Iowa/20_PR8-HY, and Hong Kong/19_PR8-HY viruses were also isolated from the tracheas of infected animals, and Hong Kong/19_PR8-HY virus was isolated from the lungs as well (Figure 2B). Thus, the PR8-HY backbone supports the replication of several recent human H3N2 viruses in Syrian hamsters.

### 3.2. The PR8-HY Backbone Enhances the Replication of Recent Human pdmH1N1 Viruses in Syrian Hamsters

**Figure 3:**
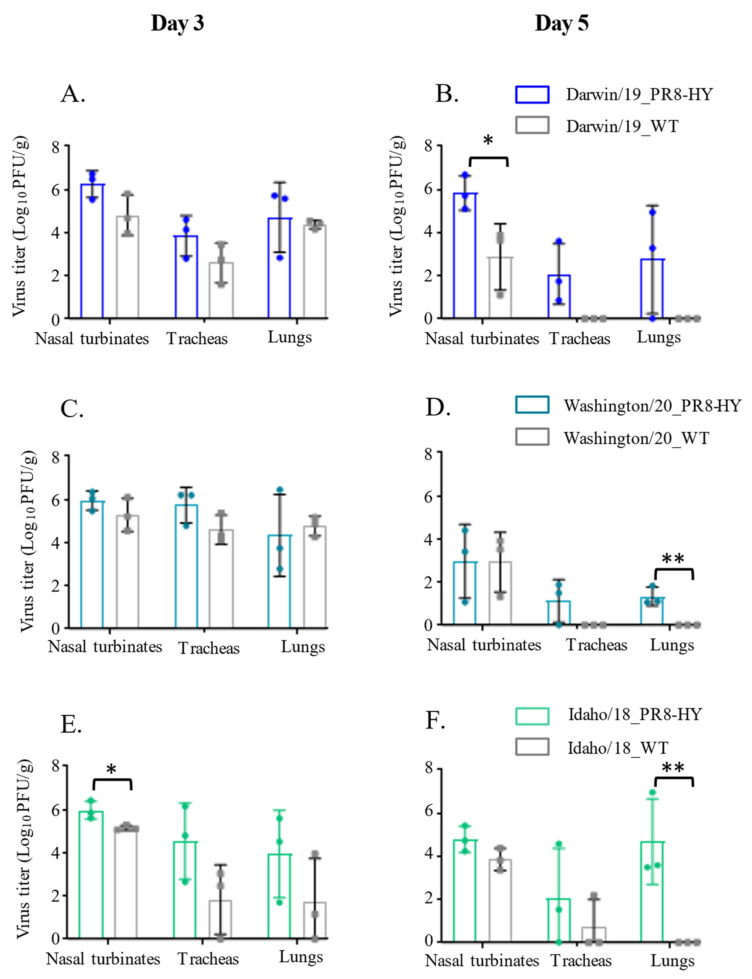
Replication of authentic and PR8-HY-based human pdmH1N1 viruses in Syrian hamsters. Six Syrian hamsters were infected with 10^6^ PFU of the indicated virus or recombinant virus with the respective HA and NA genes on the PR8-HY backbone. On days 3 and 5 p.i., three animals from each group were euthanized and virus titers in the nasal turbinates, tracheas, and lungs were determined in hCK cells. * *p* < 0.05; ** *p* < 0.01. (**A**,**C**,**E**) show the titers of the indicated viruses in hamsters on day 3 post-infection; (**B**,**D**,**F**) show the titers of the indicated viruses in hamsters on day 5 post-infection.

After demonstrating robust replication in Syrian hamsters for recombinant human H3N2 viruses with the PR8-HY backbone, we next generated three PR8-HY recombinant viruses with the HA and NA genes of three recent human pdmH1N1 viruses, which are as follows: A/Idaho/07/2018, A/Darwin/102/2019, and A/Washington/23/2020. The recombinant viruses possessing the HA and NA genes of A/Idaho/07/2018 and A/Darwin/102/2019 replicated more efficiently in vitro than the authentic viruses, but no differences were detected between the replication of the authentic and recombinant A/Washington/23/2020 viruses (Table 1). We then tested the replication of all three pairs of viruses in Syrian hamsters, as described earlier. Overall, the PR8-HY recombinant viruses replicated more efficiently in the upper and lower respiratory tract of Syrian hamsters than the authentic virus isolates, although not all of the differences were statistically significant (Figure 3A–F). On day 5 post-infection, we detected low-to-moderate virus titers in the tracheas and lungs of animals infected with the recombinant viruses, but no viruses or very low virus titers were detected in animals infected with the authentic viruses (Figure 3B,D,F). These results demonstrate that the PR8-HY backbone enhances the replication of recent human H3N2 and pdmH1N1 viruses in Syrian hamsters.

## 4. Discussion

In this study, we demonstrated that the PR8-HY backbone substantially enhances the replication of several recent human H3N2 and pdmH1N1 viruses in Syrian hamsters, compared to that of authentic virus isolates. We also detected efficient replication of recombinant recent human H3N2 viruses with the PR8-HY backbone in Syrian hamsters, although they were not tested side-by-side with authentic isolates.

Experiments in small animal models are a mainstay in influenza virus research, but authentic virus isolates may not replicate to high titers in some animal models. For example, a recent study in ferrets (the gold standard animal model for influenza virus research) had to be conducted with a very high dose (i.e., 1.3 × 10^9^ PFU) of A/Kansas/14/2017 (H3N2) of the virus [11]. Recently, we, therefore, assessed Syrian hamsters as a small animal model for influenza [3]. On epithelial cells in the pharynx, trachea, and bronchus of Syrian hamsters, we detected both α2,6- and α2,3-linked sialic acids [3]; in contrast, epithelial cells in the lungs expressed only α2,3-linked sialic acids [3]. This distribution of α2,6- and α2,3-linked sialic acids is very similar to the distribution of sialic acids in the respiratory tract of humans [12,13,14]. In accordance with these data, we previously demonstrated the replication of human H3N2 viruses (including recent isolates), human H1N1 viruses, and human influenza B viruses in the respiratory tract of Syrian hamsters, even though neither weight loss or clinical signs of disease were detected [3]. Overall, the data established Syrian hamsters as an attractive small animal model for influenza virus research. 

Here, we demonstrated that the PR8-HY backbone increased the virus titers of several recent human H3N2 and pdmH1N1 viruses compared to the authentic viruses. Influenza viruses are known to differ in their replicative ability in cultured cells and animal models. Here, we, therefore, tested a range of viruses but consistently found that the PR8-HY backbone increased virus titers. 

The limited replicative ability of some influenza viruses in animal models will result in small differences in virus titers between untreated animals and animals treated with preventative or therapeutic compounds or vaccines, thus limiting the use of those animal models. For vaccination and challenge studies or studies of novel therapeutic antibodies, the use of a recombinant virus backbone that increases virus titers in animals may, therefore, be an attractive alternative to the use of authentic virus isolates.

## Figures and Tables

**Table 1:** Virus titers of authentic and PR8-HY recombinant human H3N2 and pdmH1N1 viruses.

Original Virus	Virus Type	Subtype	Origin of Viral Genes		Virus Titer (PFU/mL)
			HA and NA Genes	Internal Genes	
A/Perth/16/2009	Authentic virus	H3N2	A/Perth/16/2009	A/Perth/16/2009	1.0 × 10^7^
	Recombinant virus	H3N2	A/Perth/16/2009	PR8-HY	4.4 × 10^8^
A/Wisconsin/15/2009	Authentic virus	H3N2	A/Wisconsin/15/2009	A/Wisconsin/15/2009	1.1 × 10^7^
	Recombinant virus	H3N2	A/Wisconsin/15/2009	PR8-HY	1.6 × 10^8^
A/Tokyo/IMS2-1/2014	Authentic virus	H3N2	A/Tokyo/IMS2-1/2014	A/Tokyo/IMS2-1/2014	1.0 × 10^7^
	Recombinant virus	H3N2	A/Tokyo/IMS2-1/2014	PR8-HY	3.7 × 10^7^
A/Darwin/102/2019	Authentic virus	pdmH1N1	A/Darwin/102/2019	A/Darwin/102/2019	1.3 × 10^7^
	Recombinant virus	pdmH1N1	A/Darwin/102/2019	PR8-HY	2.0 × 10^8^
A/Idaho/07/2018	Authentic virus	pdmH1N1	A/Idaho/07/2018	A/Idaho/07/2018	9.2 × 10^6^
	Recombinant virus	pdmH1N1	A/Idaho/07/2018	PR8-HY	7.2 × 10^8^
A/Washington/23/2020	Authentic virus	pdmH1N1	A/Washington/23/2020	A/Washington/23/2020	3.0 × 10^8^
	Recombinant virus	pdmH1N1	A/Washington/23/2020	PR8-HY	2.0 × 10^8^

## Supplementary Materials

The following supporting information can be downloaded at: https://www.mdpi.com/article/10.3390/v14081629/s1, Figure S1: Virus replication in hamsters intranasally infected with two different dosages and volumes of influenza virus.
