# Supplementary material for: Influenza Viruses Suitable for Studies in Syrian Hamsters
Source: Viruses. 2022 Jul 26;14(8):1629. doi: 10.3390/v14081629 (PMC9330595; doi:10.3390/v14081629)
Supplement: Supplementary file 1 [file viruses-14-01629-s001.zip › viruses-1838092-supplementary.pdf]

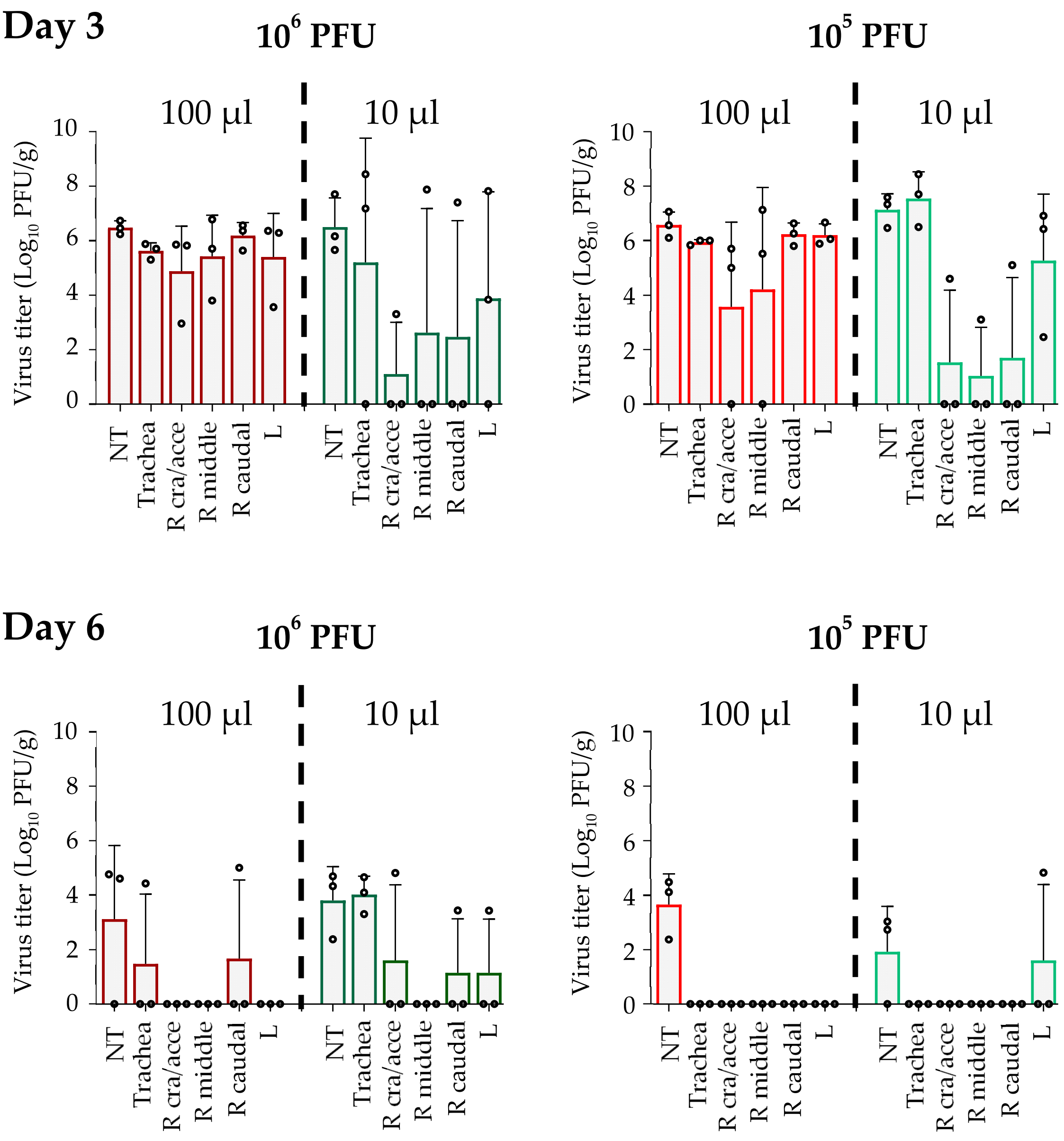

Figure S1. Virus replication in hamsters intranasally infected with two different dosages and volumes of influenza virus. Hamsters were intranasally infected with an infectious dosage of 10^6 or 10^5 PFU of A/Isumi/UT-KK001-1/2018 (pdmH1N1) virus in a volume of 100 µl or 10 µl. Lung and nasal turbinate samples were collected for virus titration on day 3 and day 6 post-infection. NT, nasal turbinate; R cra/acce, right cranial and accessory lobes; R middle, right middle lobe; R caudal, right caudal lobe; L, left lobe.
